# The associations of premorbid social isolation and social support with self-rated health and heart failure outcomes in the atherosclerosis risk in communities (ARIC) Study

**DOI:** 10.1371/journal.pone.0337517

**Published:** 2025-11-25

**Authors:** Kennedy M. Peter-Marske, Annie Green Howard, Kelly R. Evenson, Sara Jones Berkeley, Joanna Maselko, Mario Sims, Stuart D. Russell, Anna Kucharska-Newton, Kevin J. Sullivan, Wayne D. Rosamond

**Affiliations:** 1 Department of Epidemiology, Gillings School of Global Public Health, University of North Carolina at Chapel Hill, Chapel Hill, North Carolina, United States of America; 2 Department of Biostatistics, Gillings School of Global Public Health, University of North Carolina at Chapel Hill, Chapel Hill, North Carolina, United States of America; 3 Carolina Population Center, University of North Carolina at Chapel Hill, Chapel Hill, North Carolina, United States of America; 4 Department of Social Medicine, Population and Public Health, School of Medicine University of California at Riverside, Riverside, California, United States of America; 5 Division of Cardiology, Department of Medicine, Duke University School of Medicine, Durham, North Carolina, United States of America; 6 Department of Medicine, The MIND Center, University of Mississippi Medical Center, Jackson, Mississippi, United States of America; Retired-United States Environmental Protection Agency, UNITED STATES OF AMERICA

## Abstract

We assessed whether social isolation (SI), social support (SS), and subtypes of SS were associated with self-rated health trajectories and clinical heart failure (HF) outcomes among participants with incident HF hospitalizations. We included 2967 Atherosclerosis Risk in Communities study participants with incident HF hospitalization after Visit 2 (1990–1992). SI, SS, and subtypes of SS were measured at Visit 2. We identified incident HF hospitalization as ICD-9 code 428 and physician adjudicated events; on average HF occurred 17 (SD 8) years after Visit 2. We assessed associations with trajectories of annually measured self-rated health in the 4 years prior to and after incident HF hospitalization (excellent/good self-rated health on a 0–100 scale), using linear mixed effects models. We calculated hazard ratios (HR) and 95% confidence intervals (CIs) for associations with time to first all-cause rehospitalization and all-cause mortality using Cox proportional hazard models. Low overall SS had a 5.8 point (95% CI 7.8, 3.8) lower self-rated health value over time than high SS; associations of subtypes of SS with this outcome were similar. Low belonging SS was associated with greater days to first rehospitalization (HR 0.85; 95% CI 0.79, 0.96) compared to the highest tertile; however, belonging SS was not associated with mortality (HR 1.05; 95% CI 0.95, 1.17). Being socially isolated/high risk for SI was associated with greater hazard of all-cause mortality among females (HR 1.57; 95% CI 1.20, 2.06) but not males (HR 0.95; 95% CI 0.75, 1.19), compared to low SI. SI and SS were not associated with number of hospitalizations in the first year or percent of first year spent at home.

## Introduction

Poor social relationships, measured through markers such as social isolation (SI) (quantity of relationships) and low social support (SS) (quality of relationships), increase risk of heart failure (HF), as was shown in the Atherosclerosis Risk in Communities (ARIC) study [1], and result in poorer prognosis after a HF diagnosis [2–5]. Although SI and low SS have been shown to predict HF-related symptoms [6], HF self-care behaviors [7, 8] length of hospital stay [9], depressive symptoms, [4] and HF prognosis [2–5] among HF patients in various observational studies, experimental trials aimed to alter social relationships among HF patients have been largely unsuccessful in improving HF morbidity and mortality [10,11]. We hypothesized two reasons for these incongruous results. The first reason being related to later timing of intervention in terms of HF progression in experimental trials compared to earlier measurement among observational studies, as social relationships may change later in HF progression with health declines [12]. Measurement of social relationships prior to HF, hereby referred to as “premorbid”, may be more reflective of established social resources available to the patient at incident HF hospitalization, as declines in health associated with HF may positively or negatively impact social relationships or the HF patient’s perception of these relationships [12]. To our knowledge, only three observational studies have assessed the associations of premorbid social relationships with HF morbidity and mortality, yielding conflicting results [13–15]. The second reason we hypothesized for incongruous results was that there may be unmet need for interventions specific to a certain type of social relationship, of which limited types were investigated in interventions compared to observational studies.

We therefore examined premorbid SI and SS, along with specific types of SS (appraisal, belonging, self-esteem, and tangible SS), and their prospective associations with trajectories of self-rated health (SRH), and HF morbidity and mortality measured by clinical events among ARIC Study participants. Investigation of multiple social relationship aspects along with premorbid timing of the assessment may help inform and improve future interventions for HF patients.

## Methods

### Study design and population

The ARIC study [16] is a population-based prospective cohort study that recruited 15,792 middle-aged (45–64 years), predominantly Black and White males and females from four communities in the United States: Forsyth County, NC; Jackson, MS; select suburbs of Minneapolis, MN; and Washington County, MD. Participants who attended Visit 1 (1987–1989) were invited to subsequent in-person visits, of which there have been 10 to date, and were followed via annual telephone interviews (semi-annual beginning in 2012). Written informed consent was collected from all participants, and the ARIC study was approved by the following institutional review boards: University of North Carolina at Chapel Hill, Wake Forest University, University of Mississippi Medical Center, University of Minnesota, and Johns Hopkins University. De-identified data were originally accessed for this analysis in January 2023, and can be requested through the Biologic Specimen and Data Repository Information Coordinating Center (BioLINCC) website (https://biolincc.nhlbi.nih.gov/studies/aric/) after registering with the site.

Of the 14,348 participants who attended ARIC Visit 2 (1990–1992), we excluded participants who self-reported a race other than Black or White (n = 42) and Black participants from Washington County, MD or the suburbs of Minneapolis, MN (n = 49) due to small sample sizes. We further excluded any participants with prevalent HF at Visit 2 (n = 223) and those who did not experience an incident HF hospitalization during follow-up (n = 10,774). Finally, we excluded participants with missing social relationship data (n = 274) and no measures of self-rated health (n = 19), resulting in a final analysis sample size of 2,967 participants.

### Social relationships

The ARIC study used the Lubben Social Network Scale (LSNS) [17,18] to measure SI. Using 10 questions with a 0−5 rating scale, the LSNS assesses the self-reported availability of social contacts with friends, family, and peers, along with network size. Total scores ranging from 0−50 are commonly categorized according to risk for SI: low risk (≥31), moderate risk (26 –30), high risk (21 –25), and socially isolated (≤20) [19]. ARIC assessed perceived SS using the 16-item Interpersonal Support Evaluation List (ISEL), which measures total perceived SS using 16 questions from 4 subscales (appraisal, belonging, self-esteem, and tangible SS) of the original 40-item ISEL. Total possible scores ranged from 0 to 48, and subscale scores ranging from 0 to 12, with higher scores indicating greater perceived SS. There are no standard categories used for the ISEL-16.

### Heart failure definition

Cardiovascular disease events were ascertained by contacting participants annually/semi-annually, examining hospitalizations and death records from the previous year, and by using discharge lists from local hospitals and death certificates from state vital statistics offices. Since 2005, HF hospitalizations were adjudicated as definite and probable acute or chronic decompensated HF by an ARIC study physician panel according to defined criteria [20]. We defined incident HF events using ICD-9 code 428* in any position prior to 2005, and adjudicated HF hospitalizations starting in 2005, as has been done in previous studies [21]. Three different definitions of incident HF were explored as sensitivity analyses: 1) relevant ICD-9/ICD-10 codes in any position, 2) a relevant ICD-9/ICD-10 code in the primary position only, and 3) adjudicated HF hospitalizations only.

### Self-rated health (SRH) outcome

SRH is a holistic measure of perceived health that encompasses physical, mental, and social health, and is associated with risk of cardiovascular disease and all-cause mortality [22]. For this analysis, trajectories of SRH in the four years prior to and following first HF hospitalization were assessed as a participant-reported measure of perceived health status. During annual follow-up telephone interviews, ARIC participants were asked to rate their health compared to others who are the same age as excellent, good, fair, or poor. For longitudinal analysis of SRH data, we used the recommended transformation of SRH categories into a semi-continuous measure representing the probability of being “healthy”: excellent = 95, good = 80, fair = 30, poor = 15, death = 0 [23].

### Clinical outcomes

Time to first all-cause rehospitalization and number of all-cause rehospitalizations within one year post-discharge were identified using ICD-9/ICD-10 discharge codes and dates. Length of hospital stay for coronary heart disease or HF was identified using validated events by ARIC reviewer classification and was not available for any other causes of hospitalizations. We therefore defined the proportion of the first year (alive) that was spent at home as the total number of days in the first year alive minus the number of days in the first year spent in the hospital for coronary heart disease or HF, divided by the total number of days in the first year alive.

### Covariates

We identified potential confounders using a priori scientific knowledge and information from published literature. The following variables were included as covariates: sex (male or female), self-reported race + study center (MN/White, MD/White, NC/White, NC/Black, or MS/Black), employment status (homemaker, employed, unemployed, or retired), household income (≤$25,000; $25,000 - $49,999; or ≥$50,000), educational attainment (years up to 12, GED (13), 1–3 years of vocational school (14 –16), 1–4 years of college (17 –20), or graduate/professional school (21)), prior use of mental health-related medications (yes or no), age (years), marital status (married or not married), and days between Visit 2 and the incident HF hospitalization. Behavioral cardiovascular risk factors such as diet, physical activity, smoking status, and alcohol use were not included in the adjustment set as they were potential mediators on the causal pathway between social relationships and HF outcomes.

### Statistical analysis

We examined descriptive statistics of demographics, clinical measures, and social relationship variables at the time of social relationship measurement (Visit 2). Linearity and functional form for all continuous exposures and confounders were assessed by utilizing nested models and testing for differences using the likelihood ratio test, and by comparing the Akaike Information Criteria (AIC) for each nested model and for models unable to be nested. Statistical tests such as the likelihood ratio test were set to an alpha of 0.05. SS variables were modeled as categorical tertiles.

Associations of SI and SS with SRH in the 4 years before and after the incident HF hospitalization were assessed using adjusted linear mixed effects models that account for repeated measures of SRH. Participants were not required to have complete SRH data as linear mixed effects models address missing data when missingness is related to covariates included in the model. Linear mixed effects models employed an unstructured variance-covariance matrix, used maximum likelihood estimation, and included a random intercept.

Associations of social relationships with clinical measures of HF morbidity and mortality were estimated using appropriate models such as Cox proportional hazard models, Poisson models, and linear regression, all adjusted for previously outlined covariates. For Cox models, the proportional hazards assumption was assessed by visual examination of ln(-ln) survival curves for major deviations from being parallel [24]. All exposures and confounders met the proportional hazards assumption. Fine and Gray Cox proportional hazard models were used to assess the impact of death as a competing risk for time to first rehospitalization as a sensitivity analysis [25]. Participants were followed until death, loss to follow-up, or the end of follow-up used for this study (2020).

We explored whether the associations of SI, SS, and HF morbidity and mortality outcomes differed by race, sex, and marital status. We additionally assessed whether the association between SS and HF morbidity and mortality was modified by SI, as SS is somewhat determined by SI in the sense that it would be nearly impossible for a person with zero social contacts to have high social support. This was done to explore whether it was important to have low SI for better health outcomes even if an individual perceived high levels of SS. Effect measure modification was assessed by including an interaction term between the proposed modifier and the exposure in models and by stratifying analyses by the modifying factor.

We explored whether developing incident HF earlier as opposed to later impacted our results in a sensitivity analysis where we restricted the study population to participants who had incident HF within 5 years of Visit 2 (social relationship measure) to ensure both that the time between premorbid social relationships and incident HF was similar, and that follow-up times were more likely to be similar. This sensitivity analysis may also be important if social relationships are not stable over time, as studies have hypothesized that they may change in response to health declines and other major social life events [12]. In an attempt to exclude participants who may already have HF at the time of social relationship measurement, whose measures may not be true premorbid measures, we removed all participants who experienced an incident HF hospitalization within one year of Visit 2. We also conducted another sensitivity analysis using the entire analysis sample that further adjusted models for cardiovascular disease risk factors at Visit 2 such as hypertensive status, BMI, diabetes, and total cholesterol to assess whether poor baseline health status that may be associated with HF-related declines was driving associations. All analyses were conducted using SAS version 9.4 (SAS Institute, Cary, NC, United States).

## Results

### Participant characteristics

At the time of social relationship measurement, participants’ mean age was 58.5 (standard deviation (SD): 5.5) years at Visit 2 (Table 1). SI was fairly low, with 1.1% of participants classified as socially isolated and 4.6% as high risk for SI. SS was relatively high, with a median (25^th^ percentile, 75^th^ percentile (Q1 - Q3)) overall score of 38 (33 –42). The average time between Visit 2 and incident HF hospitalization (index date for time to event analyses) was 16.5 (8.2) years.

**Table 1:** Demographic, social relationship, and clinical characteristics at Visit 2 (1990-1992) of ARIC cohort participants who experienced an incident heart failure hospitalization during follow-up; N = 2967.

	N (%) or mean ± standard deviation
ARIC field center	
Forsyth Co, NC	752 (25.4)
Jackson, MS	716 (24.1)
Minneapolis, MN	700 (23.6)
Washington Co, MD	799 (26.9)
Age, years	58.5 ± 5.5
Females	1502 (50.6)
Black Americans	787 (26.5)
Education, years (Visit 1)	13.6 ± 4.4
Employment status (Visit 1)	
Homemaker	372 (12.6)
Employed	1901 (64.1)
Unemployed	79 (2.7)
Retired	613 (20.7)
*Missing*	2
Household income (Visit 1)	
Under $25,000	1139 (40.9)
$25,000 - $49,999	1048 (37.6)
Over $50,000	597 (21.4)
*Missing*	183
Married	2347 (81.3)
Living arrangement	
With spouse	2268 (76.4)
With non-spouse	379 (12.8)
Alone	320 (10.8)
Social isolation	
Isolated	32 (1.1)
High risk for isolation	136 (4.6)
Moderate risk for isolation	411 (13.9)
Low risk for isolation	2388 (80.5)
Social support, median (Q1 – Q3)	
Overall	38 (33 – 42)
Appraisal social support	10 (8 – 11 )
Belonging social support	10 (8 – 11 )
Self-esteem social support	8 (7 – 9)
Tangible social support	11 (9 – 12 )
Depression/anxiety medication use (Visit 1)	337 (11.4)
Hypertension	1160 (39.3)
Total cholesterol, mg/dL	212 ± 41
LDL cholesterol, mg/dL	136 ± 38
High cholesterol medication use	256 (8.7)
Diabetes	587 (19.9)
Body mass index, kg/m^2^	29.3 ± 6.0

Abbreviations: ARIC: Atherosclerosis Risk in Communities Study; N: number; %: percent; yr: year; Q1 – Q3: 25^th^ percentile – 75^th^ percentile; mg: milligrams; dL: deciliter; kg: kilograms; m: meter

Social isolation categories: socially isolated (8 –20), high risk (21 –25), moderate risk (26 –30), low risk (31–50)

### Social relationships and self-rated health

**Fig 1:**
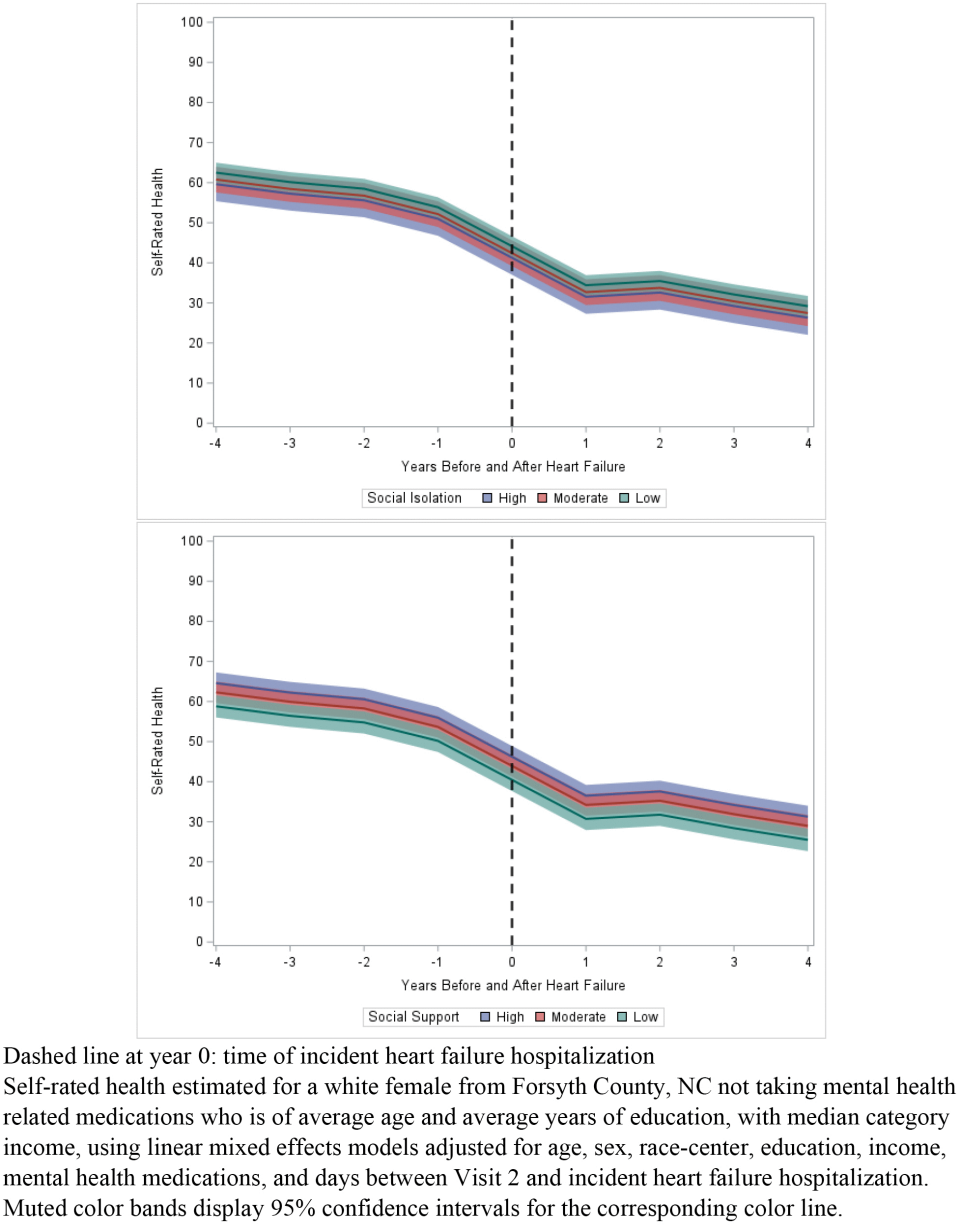
Associations of categories of social isolation and social support with trajectories of self-rated health, across the four years prior to and following incident heart failure hospitalization.

Although being in a lower category of SS or higher level of SI was associated with overall lower SRH over time, low SS and high SI were not statistically associated with a faster rate of decline in SRH over time compared to high SS and low SI respectively (Fig 1). Being socially isolated/at high-risk for isolation was associated with a −2.89 (95% confidence interval (CI): −6.38, 0.60) lower SRH value over the course of follow-up compared to those at low-risk for SI (Table 2). Being in the lowest tertile of SS was associated with poorer SRH over the course of follow-up compared to those in the highest tertile of SS as well (−5.78; 95% CI: −7.76, −3.80). Associations were greatest in magnitude for self-esteem SS and lowest in magnitude for appraisal SS.

**Table 2:** Linear mixed effect model estimated difference in self-rated health (SRH) value (0-100) associated with being at different levels of social isolation and social support (SS) over the 4 years prior to and following incident heart failure hospitalization.

	Estimated difference in SRH (95% CI)
Social isolation	
High risk	−2.89 (−6.38, 0.60)
Moderate risk	−1.72 (−4.09, 0.65)
Low risk	Referent
Overall SS	
Low	−5.78 (−7.76, −3.80)
Moderate	−2.29 (−4.24, −0.33)
High	Referent
Appraisal SS	
Low	−3.26 (−5.22, −1.29)
Moderate	0.59 (−1.35, 2.53)
High	Referent
Belonging SS	
Low	−4.58 (−6.58, −2.58)
Moderate	−2.05 (−4.00, −0.10)
High	Referent
Self-esteem SS	
Low	−5.57 (−7.39, −3.75)
Moderate	−1.61 (−3.79, 0.58)
High	Referent
Tangible SS	
Low	−4.34 (−6.30, −2.39)
Moderate	−2.16 (−4.14, −0.18)
High	Referent

CI: confidence interval.

All models adjusted for age, sex, race-center, income, years of education, the square of years of education, use of mental health medications at Visit 1, and days between Visit 2 and incident heart failure hospitalization.

Social isolation: socially isolated/high risk (8–25), moderate risk (26 –30), low risk (31–50)

Social support: low (7–34), moderate (35 –40), high (41–48)

Appraisal support: low (0–8), moderate (9 –10), high (11 –12)

Belonging support: low (1 –8), moderate (9 –10), high (11 –12)

Self-esteem support: low (0–7), moderate (8), high (9 –12)

Tangible support: low (0–9), moderate (10 –11), high (12).

Although interaction term p-values did not indicate any statistically significant differences, stratified results suggested that the association between social relationship variables and longitudinal SRH may be slightly greater in magnitude among White participants, females, and individuals who were not married (S1 Table). The interaction term p-value indicated that there were statistical differences in the association between SS and SRH over time by levels of SI (P = 0.02), namely that moderate and low SS were associated with lower SRH over time for participants who were moderately socially isolated compared to those who experienced low SI (6.98 to 7.45 points lower) (S2 Table). This trend did not apply to those who were socially isolated or at high-risk for SI, however this may be due to small sample size, as the referent category for this level of stratification only contained 13 participants.

### Social relationships and clinical measures of morbidity

Mean (SD) follow-up time was 4.8 (5.4) years after the discharge date of incident HF hospitalization (index date), and there were 14,123.5 total person-years of follow-up. The majority of participants (90%) experienced an all-cause rehospitalization after incident HF hospitalization, with a median (Q1 – Q3) time to first hospitalization of 17 (6–146) days after incident HF hospitalization. The median (Q1 – Q3) number of all-cause rehospitalizations within 1 year of incident HF hospitalization was 1 (1–2), and the median (Q1 – Q3) length of hospital stays during the first year was 5 (0–12) days. SI and SS were not statistically significantly associated with number of all-cause rehospitalizations in the first year or percent of the first year spent at home (S3 Table). Contrary to our hypothesis, being in the lowest tertile of belonging SS was statistically significantly associated with a lower hazard of all-cause rehospitalization (Hazard Ratio (HR) 0.85; 95% CI: 0.79, 0.96) compared to being in the highest tertile of belonging SS (Table 3). When using models which accounted for death as a competing risk, results were similar.

**Table 3:** Associations of social isolation and social support (SS) measured prior to heart failure with time to first all-cause rehospitalization and all-cause mortality after incident heart failure hospitalization using Cox proportional hazard models.

	Hazard Ratio (95% Confidence Interval)	
	First All-Cause Rehospitalization	All-Cause Mortality
Social isolation		
High risk	0.99 (0.84, 1.18)	1.15 (0.96, 1.37)
Moderate risk	0.96 (0.86, 1.08)	1.01 (0.89, 1.14)
Low risk	Referent	Referent
Overall SS		
Low	0.92 (0.83, 1.01)	1.05 (0.95, 1.17)
Moderate	0.93 (0.84, 1.02)	1.01 (0.91, 1.12)
High	Referent	Referent
Appraisal SS		
Low	0.96 (0.88, 1.06)	1.01 (0.91, 1.12)
Moderate	0.90 (0.81, 0.99)	0.95 (0.86, 1.05)
High	Referent	Referent
Belonging SS		
Low	0.85 (0.79, 0.96)	1.05 (0.95, 1.17)
Moderate	0.97 (0.88, 1.06)	1.01 (0.90, 1.11)
High	Referent	Referent
Self-esteem SS		
Low	0.95 (0.87, 1.04)	1.04 (0.95, 1.14)
Moderate	0.95 (0.85, 1.05)	0.96 (0.86, 1.08)
High	Referent	Referent
Tangible SS		
Low	0.97 (0.88, 1.07)	1.09 (0.98, 1.21)
Moderate	0.97 (0.88, 1.07)	1.07 (0.97, 1.19)
High	Referent	Referent

All models adjusted for age, sex, race-center, employment status, income, years of education, the square of years of education, use of mental health medications at Visit 1, and days between Visit 2 and incident heart failure hospitalization.

Social isolation: socially isolated/high risk (8–25), moderate risk (26 –30), low risk (31–50)

Social support: low (7–34), moderate (35 –40), high (41–48)

Appraisal support: low (0–8), moderate (9 –10), high (11 –12)

Belonging support: low (1 –8), moderate (9 –10), high (11 –12)

Self-esteem support: low (0–7), moderate (8), high (9 –12)

Tangible support: low (0–9), moderate (10 –11), high (12)

### Social relationships and mortality

Over the course follow-up, 2378 deaths occurred (80%) at a rate of 16.8 deaths per 100 person-years. Although belonging SS was inversely associated with time to all-cause rehospitalization, it was not statistically significantly associated with time to all-cause mortality (HR 1.05; 95% CI: 0.95, 1.17) and neither were overall SS, appraisal SS, or self-esteem SS (Table 3). Being socially isolated or at high-risk of SI was significantly associated with a higher hazard of all-cause mortality compared to participants classified as low-risk for SI among females (HR 1.57; 95% CI: 1.20, 2.06) but not males (HR 0.95; 95% CI: 0.75, 1.19) (S4 Table); this effect measure modification was supported by interaction term p-values (P = 0.02). When stratified by race, results showed a statistically significant association between low tangible SS and mortality compared to high tangible SS among White participants (HR 1.14; 95% CI: 1.01, 1.28) but not Black participants (HR 0.95; 95% CI: 0.78, 1.16); however, interaction term p-values were not statistically significant (P = 0.45). Although interaction term p-values were not statistically significant (P = 0.41), stratified results suggested that the association between being socially isolated/high risk for SI and mortality was stronger among individuals who were not married (HR 1.39; 95% CI: 1.04, 1.87) compared to those who were married (HR 1.08; 95% CI: 0.85, 1.38) (S4 Table).

### Incident HF within 5 years of social relationship measurement

When restricting the analysis sample to participants with an incident HF hospitalization within 5 years of social relationship measurement, results showed a statistically significant greater hazard of mortality associated with the lowest tertiles of overall SS (HR 1.38; 95% CI: 1.02, 1.87), appraisal SS (HR 1.33; 95% CI: 1.00, 1.78), and tangible SS (HR 1.38; 95% CI: 1.02, 1.87) compared to the highest tertiles (Table 4). It is possible that this sample included participants who were already diagnosed with HF in an outpatient setting or who had declining health due to undiagnosed HF at the time of social relationship measurement, as these participants were slightly older and displayed worse mental health and cardiovascular disease risk factors than those included in main analyses (S5 Table).

**Table 4:** Associations of social isolation and social support measured prior to heart failure with time to all-cause mortality after incident heart failure hospitalization using Cox proportional hazard models when restricting only to participants who had an incident heart failure hospitalization within 5 years of social relationship measurement (N = 332).

	N=	Hazard Ratio (95% Confidence Interval)
Social isolation		
Socially isolated/high-risk	21	1.18 (0.71, 1.96)
Moderate risk	56	0.92 (0.66, 1.29)
Low risk	255	Referent
Overall social support		
Low	128	1.38 (1.02, 1.87)
Moderate	111	1.14 (0.84, 1.54)
High	93	Referent
Appraisal support		
Low	111	1.33 (1.00, 1.78)
Moderate	111	1.08 (0.81, 1.45)
High	110	Referent
Belonging support		
Low	124	1.46 (1.09, 1.96)
Moderate	107	1.06 (0.78, 1.44)
High	101	Referent
Self-esteem support		
Low	138	1.20 (0.91, 1.57)
Moderate	67	1.08 (0.78, 1.50)
High	127	Referent
Tangible support		
Low	133	1.38 (1.02, 1.87)
Moderate	93	1.31 (0.96, 1.78)
High	106	Referent

All models adjusted for age, sex, race-center, employment status, income, years of education, the square of years of education, use of mental health medications at Visit 1, and days between Visit 2 and incident heart failure hospitalization.

Abbreviations: N: number

Social isolation: socially isolated (8 –20), high risk (21 –25), moderate risk (26 –30), low risk (31–50)

Social support: low (7–34), moderate (35 –40), high (41–48)

Appraisal support: low (0–8), moderate (9 –10), high (11 –12)

Belonging support: low (1 –8), moderate (9 –10), high (11 –12)

Self-esteem support: low (0–7), moderate (8), high (9 –12)

Tangible support: low (0–9), moderate (10 –11), high (12)

## Discussion

Our results suggested that certain aspects of premorbid social relationships may be associated with health outcomes, particularly SRH over time and all-cause mortality, among participants with HF, but that these associations my differ by factors such as race, sex, and marital status. Previous studies have shown a robust inverse association for SRH and mortality [22], which may link our findings of associations of premorbid social relationships with both of these outcomes. Lower SI and greater SS have been consistently associated with health outcomes such as prolonged years of life, and more favorable mental and physical health outcomes [26] through possible mechanisms such as behavioral and psychological processes, which in turn may impact the cardiovascular, neuroendocrine, and immune systems [27]. For HF patients particularly, social relationships may be particularly impactful on HF morbidity and mortality by providing emotional SS, help with daily living tasks, monitoring of HF symptoms, and transportation among other things [28].

Our study found greater belonging SS to be associated with a lower hazard of first all-cause rehospitalization, but did not find any association of belonging SS with greater mortality. These conflicting results may be due to the potentially complex relationship of social relationships and rehospitalizations. Although people who are socially isolated or have low SS may be in worse health and require greater hospitalizations, they may have fewer social resources to encourage them to seek rehospitalization when necessary or may have fewer tangible social resources to help them obtain medical care. Additionally, since greater SS is typically associated with favorable health behaviors, those with greater social resources may also display health-seeking behaviors such as increased healthcare utilization. Previous studies of premorbid SI/SS measures did not report an adverse association with rehospitalization measures [14,15].

Previous observational studies of premorbid social relationships with all-cause mortality after an incident HF hospitalization yielded conflicting results. The Cardiovascular Health Study found higher premorbid SI (LSNS), but not SS (ISEL), to be associated with greater mortality over 25 years of follow-up among participants who developed HF within two years of the social relationship measurements [13]. This study found no evidence of effect measure modification by sex, race, and marital status, but it may have been underpowered to assess modification as the analysis only included 529 participants. Another study examined the associations of emotional support, instrumental support, and social ties measured on average 2 years prior to incident HF hospitalization and their associations with 1-year risk of all-cause mortality, finding an association between only lower emotional support and greater mortality [14]. They found that this association existed among females but not males, partly in alignment with our finding that being socially isolated/high risk for isolation was associated with 57% higher all-cause mortality after incident HF hospitalization among females but not males.

Our results provided evidence for proximate timing of social relationship measurement to incident HF hospitalization: greater SI and lower SS of all types were associated with greater mortality when restricted to those who had an incident HF hospitalization within 5 years of exposure measurement. However, this measure of social relationships may not have been truly premorbid. These results indicate that social relationships measured at the time of HF diagnosis or shortly thereafter may be most associated with HF mortality outcomes rather than premorbid social relationships. Stability of social relationship traits and how changes in social relationships may impact HF morbidity and mortality should be the focus of future studies.

### Strengths and limitations

One major strength of this study was the premorbid assessment of social relationships, as they are less likely to have been impacted by severe HF-related health declines as social relationships measured well into HF progression [12]. Early in HF progression may also be an ideal time for intervention. However, we were limited in that we were not able to assess postmorbid social. The varying times between social relationship measurement and incident HF hospitalization allowed us to explore whether the timing of premorbid social relationship measurement in relation to incident HF hospitalization may impact results. The prevalence of SI and low SS in this sample, and in the ARIC cohort in general [29,30], was low. This may be in part due to documented differences in characteristics between the general population and cohort study participants, who tend to be overall healthier than the general population. Therefore, the associations found in this study may be underestimates of the same associations in the general population. Additionally, we were limited by low power to detect interaction effects, particularly in subsamples such as race, sex, and marital status.

To our knowledge, no previous studies have investigated the relationship between social relationships and SRH in the years before and following an incident HF hospitalization. Previously published works of premorbid social relationships with HF morbidity have primarily defined HF morbidity in terms of risk of rehospitalization within a short time period [14,15]. In order to obtain a broader understanding of clinical events related to the progression of HF, we investigated multiple morbidity and mortality outcomes. Our definition of proportion of time spent at home in the first year was limited as we only had access to length of stay for coronary heart disease and HF related hospital visits, and was therefore was likely an overestimate of the proportion of time spent at home for participants. Our main definition of incident HF hospitalization was limited in that it combined adjudicated HF after 2005 and ICD code-identified HF before adjudication began. However, based on the three sensitivity analyses defining HF in other various ways, this combinatory definition did not largely impact results. Finally, due to multiple testing, associations observed may be due to chance, although they are in agreement with various prior studies.

## Conclusions

The results of our study suggest that greater SI was associated with greater all-cause mortality among females, but not males. We also found that greater SI and lower SS of all types may be associated with poorer trajectories of SRH pre and post incident HF hospitalization. Additionally, our results suggest that social relationships measured closely to the beginning of clinically recognized HF may be the most highly associated with HF morbidity and mortality. These findings suggest that interventions among HF patients to reduce early mortality might consider an emphasis on reducing SI among females, with intervention as early as possible once the disease is clinically recognized.

## Supporting information

S1 Table Linear mixed effect model estimated difference (95% confidence interval) in self-rated health (SRH) value (0–100) associated with being at different levels of social isolation and social support (SS) over the 4 years prior to and following incident heart failure hospitalization stratified by race, sex, and marital status. (DOCX)

S2 Table Linear mixed effect model estimated difference self-rated health (SRH) associated with being at different levels of social support over the 4 years prior to and following incident heart failure hospitalization, stratified by social isolation. (DOCX)

S3 Table Associations of social support and social isolation measured prior to heart failure with number of hospitalizations within one year and percent time spent at home in the first year after incident heart failure hospitalization using Poisson regressions and multivariable linear regressions. (DOCX)

S4 Table Associations of social isolation and social support (SS) measured prior to heart failure with time to all-cause mortality after incident heart failure hospitalization using Cox proportional hazard models stratified by race, sex, and marital status. (DOCX)

S5 Table Demographic, social relationship, and clinical characteristics at Visit 2 (1990–1992) of participants who had an incident heart failure hospitalization within 5 years of social relationship measurement at Visit 2; N = 332. (DOCX)
