# Supplementary material for: The associations of premorbid social isolation and social support with self-rated health and heart failure outcomes in the atherosclerosis risk in communities (ARIC) Study
Source: PLoS One. 2025 Nov 25;20(11):e0337517. doi: 10.1371/journal.pone.0337517 (PMC12646434; doi:10.1371/journal.pone.0337517)
Supplement: S1 Table — (DOCX) [file pone.0337517.s001.docx]

| **S1 Table.** Linear mixed effect model estimated difference (95% confidence interval) in self-rated health (SRH) value (0-100) associated with being at different levels of social isolation and social support (SS) over the 4 years prior to and following incident heart failure hospitalization stratified by race, sex, and marital status | | | | | | |
| --- | --- | --- | --- | --- | --- | --- |
|  | Race | | Sex | | Marital Status | |
|  | Black | White | Male | Female | Not Married | Married |
| Social isolation |  |  |  |  |  |  |
| High risk | -2.64 (-8.55, 3.27) | -3.17 (-7.44, 1.09) | -2.30 (-6.82, 2.22) | -4.10 (-9.59, 1.39) | -2.85 (-8.33, 2.64) | -4.37 (-9.21, 0.46) |
| Moderate risk | -1.81 (-6.07, 2.45) | -1.71 (-4.54, 1.11) | -1.30 (-4.60, 2.01) | -2.53 (-5.93, 0.88) | -2.18 (-6.72, 2.36) | -1.79 (-4.74, 1.13) |
| Low risk | Referent | Referent | Referent | Referent | Referent | Referent |
| Overall SS |  |  |  |  |  |  |
| Low | -3.12 (-6.78, 0.55) | -6.63 (-8.90, -4.27) | -4.45 (-7.32, -1.57) | -7.18 (-9.94, -4.41) | -8.33 (-12.89, -3.77) | -4.94 (-7.19, -2.69) |
| Moderate | -1.26 (-5.13, 2.61) | -2.60 (-4.86, -0.34) | -1.95 (-4.78, 0.88) | -2.55 (-5.25, 0.15) | -6.35 (-11.00, -1.70) | -1.29 (-3.47, 0.89) |
| High | Referent | Referent | Referent | Referent | Referent | Referent |
| Appraisal SS |  |  |  |  |  |  |
| Low | -2.31 (-6.07, 1.45) | -3.46 (-5.76, -1.16) | -3.12 (-5.89, -0.36) | -3.52 (-6.33, -0.70) | -6.22 (-10.78, -1.66) | -2.65 (-4.86, -0.44) |
| Moderate | 1.33 (-2.34, 4.99) | 0.63 (-1.66, 2.91) | 0.27 (-2.58, 3.12) | 1.17 (-1.48, 3.38) | 0.81 (-3.64, 5.24) | 0.70 (-1.49, 2.88) |
| High | Referent | Referent | Referent | Referent | Referent | Referent |
| Belonging SS |  |  |  |  |  |  |
| Low | -3.39 (-7.22, 0.44) | -4.86 (-7.21, -2.51) | -2.97 (-5.85, -0.09) | -6.29 (-9.11, -3.47) | -7.56 (-12.21, -2.91) | -3.64 (-5.89, -1.39) |
| Moderate | -0.75 (-4.56, 3.06) | -2.30 (-4.58, -0.02) | -1.13 (-3.99, 1.74) | -2.98 (-5.66, -0.30) | -1.51 (-6.02, 3.01) | -1.88 (-4.08, 0.31) |
| High | Referent | Referent | Referent | Referent | Referent | Referent |
| Self-esteem SS |  |  |  |  |  |  |
| Low | -3.78 (-7.17, -0.39) | -6.21 (-8.37, -4.05) | -3.52 (-6.18, -0.87) | -7.44 (-9.94, -4.95) | -6.33 (-10.49, -2.16) | -5.02 (-7.08, -2.96) |
| Moderate | -3.04 (-7.52, 1.44) | -1.46 (-3.98, 1.06) | -0.03 (-3.03, 2.96) | -3.32 (-6.54, -0.11) | -4.84 (-10.20, 0.52) | -0.44 (-2.87, 1.99) |
| High | Referent | Referent | Referent | Referent | Referent | Referent |
| Tangible SS |  |  |  |  |  |  |
| Low | -1.88 (-5.44, 1.68) | -5.03 (-7.35, -2.70) | -2.74 (-5.56, 0.08) | -5.93 (-8.64, -3.22) | -6.34 (-10.74, -1.95) | -3.58 (-5.80, -1.36) |
| Moderate | -5.10 (-9.14, -1.05) | -1.40 (-3.68, 0.87) | -2.10 (-4.88, 0.68) | -2.60 (-5.43, 0.23) | -6.26 (-11.13, -1.40) | -1.21 (-3.42, 1.00) |
| High | Referent | Referent | Referent | Referent | Referent | Referent |
| All models adjusted for age, sex, race-center, income, years of education, the square of years of education, use of mental health medications at Visit 1, and days between Visit 2 and incident heart failure hospitalization.  Models stratified by race do not adjust for race, those stratified by sex do not adjust for sex, and those stratified by marital status to not adjust for marital status.  Social isolation: socially isolated/high risk (8 – 25), moderate risk (26 – 30), low risk (31 – 50)  Social support: low (7-34), moderate (35-40), high (41-48)  Appraisal support: low (0-8), moderate (9-10), high (11-12)  Belonging support: low (1-8), moderate (9-10), high (11-12)  Self-esteem support: low (0-7), moderate (8), high (9-12)  Tangible support: low (0-9), moderate (10-11), high (12) | | | | | | |
