# Supplementary material for: The associations of premorbid social isolation and social support with self-rated health and heart failure outcomes in the atherosclerosis risk in communities (ARIC) Study
Source: PLoS One. 2025 Nov 25;20(11):e0337517. doi: 10.1371/journal.pone.0337517 (PMC12646434; doi:10.1371/journal.pone.0337517)
Supplement: S2 Table — (DOCX) [file pone.0337517.s002.docx]

| **S2 Table.** Linear mixed effect model estimated difference self-rated health (SRH) associated with being at different levels of social support over the 4 years prior to and following incident heart failure hospitalization, stratified by social isolation | | | |
| --- | --- | --- | --- |
|  | Estimated difference in SRH value  (95% confidence interval) | | |
|  | Social support | | |
| Social isolation | Low | Moderate | High |
| Socially isolated/high-risk | -5.54 (-19.14, 8.07) | -12.86 (-27.40, 1.67) | Referent |
| Moderate-risk | -12.56 (-18.97, -6.15) | -8.27 (-15.26, -1.28) | Referent |
| Low-risk | -5.11 (-7.41, -2.80) | -1.29 (-3.38, 0.84) | Referent |
| All models adjusted for age, sex, race-center, income, years of education, the square of years of education, use of mental health medications at Visit 1, and days between Visit 2 and incident heart failure hospitalization.  Social isolation: socially isolated (8 – 20), high risk (21 – 25), moderate risk (26 – 30), low risk (31 – 50)  Social support: low (7-34), moderate (35-40), high (41-48) | | | |
