# Supplementary material for: The associations of premorbid social isolation and social support with self-rated health and heart failure outcomes in the atherosclerosis risk in communities (ARIC) Study
Source: PLoS One. 2025 Nov 25;20(11):e0337517. doi: 10.1371/journal.pone.0337517 (PMC12646434; doi:10.1371/journal.pone.0337517)
Supplement: S3 Table — (DOCX) [file pone.0337517.s003.docx]

| **S3 Table.** Associations of social support and social isolation measured prior to heart failure with number of hospitalizations within one year and percent time spent at home in the first year after incident heart failure hospitalization using Poisson regressions and multivariable linear regressions | | |
| --- | --- | --- |
|  | Number of Hospitalizations  Mean (95% Confidence Interval) | Percent Time at Home  Difference (95% Confidence Interval) |
| Social isolation |  |  |
| Socially isolated/high-risk | 2.73 (1.65, 4.49) | -0.96 (-3.93, 2.02) |
| Moderate risk | 2.80 (1.68, 4.68) | -1.26 (-5.68, 3.17) |
| Low risk | 2.49 (1.50, 4.13) | Referent |
| Overall social support |  |  |
| Low | 2.68 (1.63, 4.42) | -0.34 (-2.85, 2.17) |
| Moderate | 2.67 (1.61, 4.42) | -0.43 (-2.90, 2.03) |
| High | 2.74 (1.66, 4.53) | Referent |
| Appraisal support |  |  |
| Low | 2.67 (1.62, 4.40) | -0.49 (-2.96, 1.98) |
| Moderate | 2.71 (1.64, 4.48) | -0.77 (-3.22, 1.68) |
| High | 2.72 (1.65, 4.50) | Referent |
| Belonging support |  |  |
| Low | 2.66 (1.61, 4.38) | -1.55 (-4.07, 0.97) |
| Moderate | 2.71 (1.64, 4.47) | -0.44 (-2.90, 2.01) |
| High | 2.77 (1.68, 4.58) | Referent |
| Self-esteem support |  |  |
| Low | 2.71 (1.65, 4.47) | 0.91 (-1.40, 3.21) |
| Moderate | 2.63 (1.59, 4.35) | 0.55 (-2.21, 3.31) |
| High | 2.71 (1.64, 4.47) | Referent |
| Tangible support |  |  |
| Low | 2.70 (1.64, 4.45) | -0.87 (-3.34, 1.60) |
| Moderate | 2.65 (1.60, 4.38) | -0.50 (-3.01, 2.00) |
| High | 2.67 (1.62, 4.42) | Referent |
| All models adjusted for age, sex, race-center, employment status, income, years of education, the square of years of education, use of mental health medications at Visit 1, and days between Visit 2 and incident heart failure hospitalization.  Social isolation: socially isolated (8 – 20), high risk (21 – 25), moderate risk (26 – 30), low risk (31 – 50)  Social support: low (7-34), moderate (35-40), high (41-48)  Appraisal support: low (0-8), moderate (9-10), high (11-12)  Belonging support: low (1-8), moderate (9-10), high (11-12)  Self-esteem support: low (0-7), moderate (8), high (9-12)  Tangible support: low (0-9), moderate (10-11), high (12) | | |
