# Supplementary material for: The associations of premorbid social isolation and social support with self-rated health and heart failure outcomes in the atherosclerosis risk in communities (ARIC) Study
Source: PLoS One. 2025 Nov 25;20(11):e0337517. doi: 10.1371/journal.pone.0337517 (PMC12646434; doi:10.1371/journal.pone.0337517)
Supplement: S4 Table — (DOCX) [file pone.0337517.s004.docx]

| **S4 Table.** Associations of social isolation and social support (SS) measured prior to heart failure with time to all-cause mortality after incident heart failure hospitalization using Cox proportional hazard models stratified by race, sex, and marital status | | | | | | |
| --- | --- | --- | --- | --- | --- | --- |
|  | Hazard Ratio (95% Confidence Interval) | | | | | |
|  | Race | | Sex | | Marital Status | |
|  | Black | White | Male | Female | Not Married | Married |
| Social isolation |  |  |  |  |  |  |
| High risk | 1.21 (0.88, 1.67) | 1.12 (0.91, 1.38) | 0.95 (0.75, 1.19) | 1.57 (1.20, 2.06) | 1.39 (1.04, 1.87) | 1.08 (0.85, 1.38) |
| Moderate risk | 1.09 (0.86, 1.38) | 0.97 (0.84, 1.12) | 0.97 (0.82, 1.14) | 1.06 (0.88, 1.27) | 1.02 (0.80, 1.31) | 1.00 (0.86, 1.17) |
| Low risk | Referent | Referent | Referent | Referent | Referent | Referent |
| Overall SS |  |  |  |  |  |  |
| Low | 0.93 (0.76, 1.14) | 1.09 (0.96, 1.23) | 1.00 (0.86, 1.16) | 1.12 (0.96, 1.30) | 1.11 (0.86, 1.42) | 1.03 (0.92, 1.16) |
| Moderate | 0.98 (0.80, 1.21) | 1.02 (0.90, 1.14) | 1.01 (0.87, 1.17) | 1.02 (0.88, 1.18) | 1.00 (0.78, 1.29) | 1.00 (0.89, 1.12) |
| High | Referent | Referent | Referent | Referent | Referent | Referent |
| Appraisal SS |  |  |  |  |  |  |
| Low | 0.97 (0.79, 1.19) | 1.01 (0.90, 1.13) | 1.00 (0.87, 1.15) | 1.02 (0.87, 1.18) | 1.04 (0.81, 1.33) | 0.99 (0.88, 1.11) |
| Moderate | 1.06 (0.87, 1.30) | 0.90 (0.80, 1.01) | 0.98 (0.85, 1.13) | 0.92 (0.80, 1.06) | 0.89 (0.70, 1.13) | 0.93 (0.83, 1.04) |
| High | Referent | Referent | Referent | Referent | Referent | Referent |
| Belonging SS |  |  |  |  |  |  |
| Low | 0.95 (0.77, 1.18) | 1.08 (0.96, 1.22) | 1.00 (0.87, 1.16) | 1.11 (0.96, 1.30) | 1.15 (0.89, 1.48) | 1.02 (0.90, 1.14) |
| Moderate | 0.92 (0.74, 1.14) | 1.02 (0.91, 1.15) | 0.95 (0.82, 1.10) | 1.08 (0.93, 1.25) | 1.04 (0.82, 1.33) | 0.99 (0.88, 1.11) |
| High | Referent | Referent | Referent | Referent | Referent | Referent |
| Self-esteem SS |  |  |  |  |  |  |
| Low | 0.99 (0.83, 1.20) | 1.04 (0.93, 1.16) | 0.97 (0.85, 1.11) | 1.12 (0.98, 1.28) | 1.21 (0.97, 1.50) | 0.98 (0.88, 1.09) |
| Moderate | 1.07 (0.84, 1.38) | 0.93 (0.82, 1.06) | 0.98 (0.84, 1.15) | 0.95 (0.79, 1.13) | 1.02 (0.76, 1.36) | 0.91 (0.80, 1.04) |
| High | Referent | Referent | Referent | Referent | Referent | Referent |
| Tangible SS |  |  |  |  |  |  |
| Low | 0.95 (0.78, 1.16) | 1.14 (1.01, 1.28) | 1.10 (0.95, 1.27) | 1.10 (0.95, 1.27) | 1.06 (0.84, 1.35) | 1.08 (0.97, 1.22) |
| Moderate | 1.15 (0.92, 1.44) | 1.06 (0.94, 1.19) | 1.02 (0.89, 1.18) | 1.13 (0.97, 1.32) | 1.25 (0.96, 1.63) | 1.03 (0.92, 1.16) |
| High | Referent | Referent | Referent | Referent | Referent | Referent |
| All models adjusted for age, sex, race-center, employment status, income, years of education, the square of years of education, use of mental health medications at Visit 1, and days between Visit 2 and incident heart failure hospitalization.  Models stratified by race do not adjust for race, those stratified by sex do not adjust for sex, and those stratified by marital status to not adjust for marital status.  Social isolation: socially isolated/high risk (8 – 25), moderate risk (26 – 30), low risk (31 – 50)  Social support: low (7-34), moderate (35-40), high (41-48)  Appraisal support: low (0-8), moderate (9-10), high (11-12)  Belonging support: low (1-8), moderate (9-10), high (11-12)  Self-esteem support: low (0-7), moderate (8), high (9-12)  Tangible support: low (0-9), moderate (10-11), high (12) | | | | | | |
