# Supplementary material for: The associations of premorbid social isolation and social support with self-rated health and heart failure outcomes in the atherosclerosis risk in communities (ARIC) Study
Source: PLoS One. 2025 Nov 25;20(11):e0337517. doi: 10.1371/journal.pone.0337517 (PMC12646434; doi:10.1371/journal.pone.0337517)
Supplement: S5 Table — (DOCX) [file pone.0337517.s005.docx]

| **S5 Table.** Demographic, social relationship, and clinical characteristics at Visit 2 (1990-1992) of participants who had an incident heart failure hospitalization within 5 years of social relationship measurement at Visit 2; N=332 | |
| --- | --- |
|  | N (%) or mean ± standard deviation |
| ARIC field center |  |
| Forsyth Co, NC | 65 (19.6) |
| Jackson, MS | 96 (28.9) |
| Minneapolis, MN | 72 (21.7) |
| Washington Co, MD | 99 (29.8) |
| Age, years | 60.0 ± 5.1 |
| Females | 136 (41.0) |
| Black Americans | 104 (31.3) |
| Education, years (Visit 1) | 12.6 ± 4.6 |
| Employment status (Visit 1) |  |
| Homemaker | 39 (11.8) |
| Employed | 167 (50.3) |
| Unemployed | 13 (3.9) |
| Retired | 113 (34.0) |
| Household income (Visit 1) |  |
| Under $25,000 | 162 (52.3) |
| $25,000 - $49,999 | 100 (32.3) |
| Over $50,000 | 48 (15.5) |
| *Missing* | 22 |
| Married | 235 (72.8) |
| Living arrangement |  |
| With spouse | 230 (69.3) |
| With non-spouse | 63 (19.0) |
| Alone | 39 (11.8) |
| Social isolation |  |
| Isolated | 5 (1.5) |
| High risk for isolation | 16 (4.8) |
| Moderate risk for isolation | 56 (16.9) |
| Low risk for isolation | 255 (76.8) |
| Social support, median (Q1 – Q3) |  |
| Overall | 37 (32 – 41) |
| Appraisal social support | 9 (8 – 11) |
| Belonging social support | 9 (8 – 11) |
| Self-esteem social support | 8 (6 – 9) |
| Tangible social support | 10 (8 – 12) |
| Depression/anxiety medication use (Visit 1) | 43 (13.0) |
| Hypertension | 162 (49.1) |
| Total cholesterol, mg/dL | 214 ± 49 |
| LDL cholesterol, mg/dL | 137 ± 40 |
| High cholesterol medication use | 27 (8.2) |
| Diabetes | 116 (35.1) |
| Body mass index, kg/m^2^ | 30.1 ± 6.9 |
| Abbreviations: ARIC: Atherosclerosis Risk in Communities study; N: number; %: percent; yr: year, Q1 – Q3: 25^th^ percentile – 75^th^ percentile; mg: milligrams; dL: deciliter; kg: kilograms; m: meter  Social isolation categories: socially isolated (8 – 20), high risk (21 – 25), moderate risk (26 – 30), low risk (31 – 50) | |
